# Postmortem Oxycodone Toxicology: A Systematic Review and Meta-Analysis of Concentrations and Interpretative Markers

**DOI:** 10.3390/molecules31122051

**Published:** 2026-06-11

**Authors:** Maria Sofia Fede, Manuela Pellegrini, Adele Minutillo, Alida Likey, Angelo Montana, Francesco Paolo Busardò, Anastasio Tini

**Affiliations:** 1 Section of Legal Medicine, Department of Biomedical Sciences and Public Health, Marche Polytechnic University, 60126 Ancona, Italy; mariasofiafede13@gmail.com (M.S.F.); alidalikey@gmail.com (A.L.); angelomontana49@gmail.com (A.M.); anastasiotini78@gmail.com (A.T.); 2 National Center on Addiction and Doping, National Institute of Health, 00161 Rome, Italy; manuela.pellegrini@iss.it (M.P.); adele.minutillo@iss.it (A.M.); 3 Maria Cecilia Hospital, GVM Care & Research, 48033 Cotignola, Italy

**Keywords:** postmortem oxycodone toxicology, systematic review, meta-analysis, oxycodone-related fatalities, peripheral blood concentrations, oxycodone metabolites, alternative matrices, LC–MS/MS

## Abstract

**Background**: Oxycodone is a widely prescribed semi-synthetic opioid central to pain management. However, establishing its role in death when detected in postmortem toxicology is challenging. Quantitative evidence to support forensic interpretation remains limited. **Methods**: A systematic review and meta-analysis was conducted following PRISMA 2020 guidelines. PubMed and Scopus were searched through 3 March 2026, for studies reporting quantitative postmortem oxycodone concentrations in human biological matrices. Peripheral blood was predefined as the primary matrix for quantitative synthesis. Random-effects meta-analysis with restricted maximum likelihood estimation was performed on logarithmically transformed concentrations to compare fatal intoxications versus non-intoxication deaths and mono- versus mixed-intoxication cases. Pooled estimates were reported as geometric mean concentrations with 95% confidence and prediction intervals. Secondary analyses evaluated metabolite-to-parent ratios, alternative matrices, and postmortem interval (PMI). **Results**: Twenty-three studies comprising 4335 oxycodone-positive decedents were included in the qualitative synthesis, and 14 studies in the quantitative meta-analysis. Fatal intoxication cases (*n* = 1555) showed a pooled geometric mean peripheral blood oxycodone concentration of 0.37 mg/L (95% CI: 0.24–0.58; I^2^ = 93.5%), compared with 0.08 mg/L (95% CI: 0.04–0.15; I^2^ = 98.5%) in non-intoxication deaths (*n* = 1409). Mono-intoxication cases (*n* = 135) exhibited higher concentrations (0.52 mg/L; 95% CI: 0.22–1.21; I^2^ = 82.3%) than mixed-drug fatalities (*n* = 511; 0.29 mg/L; 95% CI: 0.13–0.65; I^2^ = 93.1%). Metabolite data indicated that noroxycodone and oxymorphone patterns may assist in distinguishing acute intake and metabolic variability. Alternative matrices, particularly vitreous humor and solid tissues provided complementary interpretative information, while PMI contributed concentration variability. **Conclusions**: The key quantitative findings of this meta-analysis indicate higher peripheral blood oxycodone levels in fatal intoxications than in non-intoxication deaths. However, substantial heterogeneity precludes the definition of absolute concentration cut-offs, emphasizing the need to approach postmortem oxycodone interpretation within a probabilistic forensic framework integrating circumstantial evidence, sampling time, metabolite ratios, and data from alternative biological matrices.

## 1. Introduction

Oxycodone (14-hydroxy-7,8-dihydrocodeinone) is a semi-synthetic opioid agonist derived from thebaine. It acts primarily as an agonist at the μ-opioid receptor, with weaker activity at κ and δ receptors, producing potent central analgesia. Structural modifications compared with morphine, particularly the presence of a 14-hydroxyl group and a 6-keto function, contribute to its pharmacokinetic profile, including enhanced oral bioavailability (60–80%), moderate plasma protein binding, lipophilicity, and a volume of distribution of 2–5 L/kg [1]. These properties, along with efficient blood–brain barrier permeability, support its widespread clinical use in moderate to severe pain management, including cancer-related pain, postoperative settings, and chronic pain syndromes [2]. Experimental evidence also suggests potential cardioprotective effects in myocardial ischemia–reperfusion injury, highlighting a complex pharmacodynamic profile with both clinical and forensic implications [3,4]. In clinical practice, oxycodone is available in multiple formulations, including immediate-release (IR) and controlled-release (CR) oral preparations, as well as parenteral formulations for intravenous or subcutaneous administration in selected cases [5]. It undergoes extensive hepatic metabolism predominantly via cytochrome P450–mediated oxidative pathways. Phase I metabolism is dominated by N-demethylation to noroxycodone via CYP3A4, while O-demethylation to oxymorphone, mediated by CYP2D6, represents a quantitatively minor but pharmacologically relevant pathway due to its higher μ-receptor affinity than morphine and its approximately 14-fold greater potency than oxycodone [6]. Further biotransformations generate noroxymorphone and other minor reduced metabolites that, although limited in opioid activity, contribute to the analytical profile of oxycodone in biological matrices, while glucuronide conjugates formed via UGT enzymes represent biomarkers of systemic exposure [7]. Opioid-related mortality is a significant public health concern, with substantial regional variation. In the United States, nearly 80,000 opioid-related overdose deaths occurred in 2023, largely driven by high-potency synthetic opioids [8]. In Europe, although heroin and synthetic opioids remain significant contributors, a substantial proportion of opioid-related harm is associated with the therapeutic use, misuse, and diversion of prescription opioids, including oxycodone [9]. Consequently, the frequent detection of oxycodone in overdose deaths—often in combination with other central nervous system depressants—poses significant challenges for forensic interpretation. Postmortem drug concentrations may be influenced by numerous variables, including dose, route of administration, tolerance, metabolic variability, drug stability, and postmortem redistribution, thereby limiting the direct interpretation of isolated concentration values [10]. The substantial overlap between therapeutic, toxic, and fatal concentration ranges further complicates interpretation and highlights the need for comprehensive forensic and circumstantial evaluation [11]. Within this framework, postmortem toxicology plays a crucial role not only in detecting oxycodone but also in identifying the most informative biological matrices. Peripheral blood remains the reference specimen in forensic toxicology; however, alternative matrices —including central blood, vitreous humor, gastric contents, urine, bile, pericardial fluid, and solid tissues such as brain, liver, and skeletal muscle— may provide complementary information, particularly when peripheral blood is unavailable, degraded, or potentially affected by postmortem redistribution [12,13]. This analytical complexity underscores the need for highly sensitive and specific methodologies capable of accurately quantifying both the parent compound and its metabolites across multiple matrices, while enabling evaluation of metabolite-to-parent ratios and postmortem redistribution phenomena [14]. Despite the growing number of case reports and small case series, harmonized and systematically synthesized data integrating postmortem oxycodone concentration distributions with complementary forensic interpretative markers remain limited, and no dedicated meta-analyses are currently available. The only existing review addressing postmortem reference drug concentrations is a non-systematic summary broadly covering new synthetic opioids, without a specific focus on oxycodone or quantitative pooling of concentration data [14]. In this context, the present systematic review and meta-analysis aimed to provide the first dedicated quantitative synthesis of postmortem oxycodone concentrations integrated with alternative matrices, metabolite ratios, pharmacogenomic variability, and postmortem interval (PMI)-related factors, thereby contributing to the standardization of postmortem oxycodone interpretation and supporting complex death investigations through improved analytical and medico-legal assessment.

## 2. Materials and Methods

### 2.1. Protocol Data Sources and Search Strategy

This systematic review and meta-analysis was conducted in accordance with the PRISMA 2020 guidelines [15], and the PRISMA checklist is provided as Supplementary Material (Table S1). The study protocol, including eligibility criteria, outcomes, and statistical methods, was defined a priori. A comprehensive literature search was independently performed by two authors using PubMed (MEDLINE) and Scopus, with no restrictions on publication year. The final search was conducted on 3 March 2026. The search targeted postmortem studies reporting quantitative concentrations of oxycodone and its major metabolites in human biological matrices. Peripheral femoral blood was pre-specified as the primary matrix for quantitative meta-analysis, while data from other matrices were included quantitatively when available, and matrix-to-peripheral blood ratios were calculated to provide comparative insights. The complete search string developed for PubMed included: (“Oxycodone” [Mesh] OR oxycodone OR noroxycodone OR oxymorphone OR noroxymorphone) AND (postmortem OR “post-mortem” OR autopsy OR “drug-related death” OR overdose OR poisoning OR fatal) AND (“peripheral blood” OR “femoral blood” OR “central blood” OR “cardiac blood” OR blood OR “vitreous humor” OR urine OR “gastric content” OR “intraosseous fluid” OR brain OR liver OR kidney OR muscle OR adipose OR bone) AND (“LC-MS/MS” OR “liquid chromatography tandem mass spectrometry” OR “gas chromatography” OR GC-MS OR LC-MS OR quantification OR concentration). This search strategy was adapted for the Scopus database, and the corresponding query is provided in the Supplementary Material.

### 2.2. Selection Criteria, Data Extraction, and Outcomes

Studies were selected according to predefined eligibility criteria. Only original human postmortem studies in which oxycodone was explicitly investigated were included, even in the context of mixed drug intoxications. Eligible studies for the qualitative synthesis included those reporting postmortem oxycodone data in biological matrices, including alternative matrices and metabolite ratios. For the quantitative meta-analysis, only studies providing peripheral blood concentrations stratified by cause of death (fatal intoxication vs. non-intoxication deaths) were included. Studies with unclear or insufficiently reliable intoxication/non-intoxication classification were retained for qualitative synthesis but excluded from quantitative pooling. Exclusion criteria comprised animal studies, review articles, systematic reviews or meta-analyses, and non-English publications. After duplicate removal, titles and abstracts were independently screened by two authors. Records not meeting the inclusion criteria were excluded. Full-text articles were then assessed for eligibility. Data extracted included study design, population characteristics (age, sex, sample size), biological matrix, analytical methods and lower limits of quantification (LLOQs), oxycodone concentrations, cause-of-death classification, and when available postmortem interval, metabolite-to-parent ratios, pharmacogenomic data, and autopsy findings. Methodological quality and potential risk of bias were assessed using a structured domain-based framework considering case definition and representativeness, completeness of concentration reporting, analytical validation, limits of detection/quantification, and potential confounding factors (e.g., polypharmacy). These domains were evaluated in terms of their possible impact on concentration estimates, forensic interpretation, and between-study comparability. The primary outcome was the quantitative synthesis of postmortem peripheral blood oxycodone concentrations in fatal intoxication versus non-intoxication deaths. Planned subgroup analyses distinguished mono-intoxications (oxycodone-only) from mixed-drug intoxication cases. Studies not eligible for quantitative pooling were included in the qualitative synthesis to support contextual forensic interpretation. The data supporting the findings of this study are available from the corresponding author upon reasonable request.

### 2.3. Statistical Analysis

Continuous data available as individual concentration values or directly reported means and standard deviations (SDs) were retained whenever available in the original studies. Medians with ranges, interquartile ranges (IQRs), or percentiles were converted to means and SDs using established methods (Wan et al.). In the presence of multiple summary measures, percentile- or IQR-based estimates were preferentially used over minimum–maximum ranges because they are less influenced by extreme values and more appropriate for highly skewed toxicological data. For studies contributing multiple subgroups to the same meta-analytic category, subgroup estimates were combined using weighted means and pooled variances before inclusion in the corresponding analysis. Given the strictly positive and markedly right-skewed nature of postmortem toxicological concentrations, quantitative syntheses were performed on the natural logarithmic scale. Meta-analyses were conducted using random-effects models with restricted maximum likelihood estimation (REML), given the expected clinical, analytical, and methodological heterogeneity across studies [16]. Pooled estimates were subsequently back-transformed and reported as geometric mean (GM) concentrations with 95% confidence intervals and prediction intervals. Heterogeneity was assessed using the I^2^ statistic and τ^2^ estimates [17]. Subgroup analyses were performed according to cause-of-death classification (fatal intoxication vs. non-intoxication deaths) and intoxication pattern (mono-intoxication vs. mixed-drug intoxication). Publication bias was not formally assessed because the limited number of studies and the substantial between-study heterogeneity would not allow reliable interpretation of funnel plot asymmetry or related statistical tests. All analyses were performed in R (R Foundation for Statistical Computing, Vienna, Austria) version 4.5.3 using the meta package, with significance set at *p* < 0.05.

## 3. Results

### 3.1. Characteristics of Studies Included in the Systematic Review and Meta-Analysis

**Figure 1:**
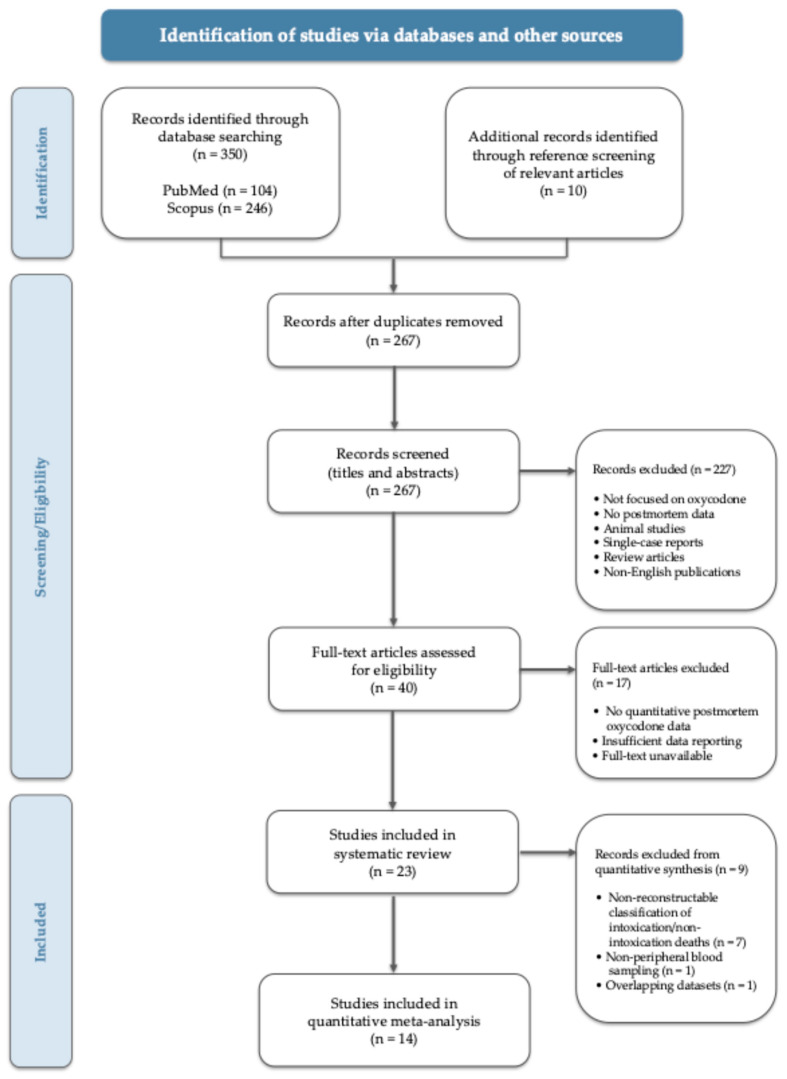
PRISMA 2020 flow diagram illustrating the study selection process for the systematic review and meta-analysis.

The detailed study selection process is summarized in the PRISMA flow diagram (Figure 1). The systematic search identified 350 records (104 from PubMed and 246 from Scopus) and one additional relevant study was identified through reference screening. After removing duplicates, titles and abstracts of 267 records were screened, and 40 full-text articles were assessed for eligibility. Following full-text evaluation, 23 studies met the inclusion criteria for qualitative synthesis. Of these, 14 studies provided sufficient peripheral blood concentration data allowing classification according to cause of death (fatal intoxication vs. non-intoxication deaths) and were therefore included in the quantitative meta-analysis [11,13,18,19,20,21,22,23,24,25,26,27,28,29]. The remaining studies were retained for analysis of alternative matrices, metabolite ratios, or pharmacogenomic insights [30,31,32,33,34,35,36,37,38]. All included studies were observational in design. Study characteristics, including population demographics, analytical methodologies, and sampled matrices, are summarized in Table 1. Toxicological results, including PMI, concentrations of oxycodone and its metabolites, co-intoxicants, and relevant postmortem findings, are reported in Table 2.

### 3.2. Population Demographics

A total of 4335 decedents with postmortem oxycodone detected were evaluated. Sample sizes varied widely, from small case series [18,33] to cohorts exceeding 800 cases [19,37]. Over a temporal period from 1992 to 2025, populations represented multiple countries, primarily the USA (*n* = 10), Nordic countries (*n* = 9: Sweden 5, Finland 2, Norway and Denmark 1 each), as well as Australia (*n* = 2), Italy, and Scotland (one study each). Most studies reported a male predominance (median of 65%) and mean ages ranging from 23 to 68 years, with most studies between 39 and 57 years (overall mean 46 years, with a median of 44 years).

### 3.3. Analytical Methods and LLOQ for Oxycodone

Oxycodone concentrations were determined using a variety of analytical methods. Immunoassays (e.g., ELISA, radioimmunoassay) were commonly used for screening [24,25,26,37,38], complemented by UPLC–MS/MS [18,30] and LC–TOF-MS [21,33]. Confirmation techniques mainly involved LC–MS/MS, GC-MS, or combinations such as GC–MS/MS and LC–MS/MS, often preceded by sample preparation methods like solid-phase extraction (SPE) or protein precipitation to improve quantitative accuracy. The accuracy of quantitative results depends heavily on these techniques, which is particularly critical when analyzing complex or decomposed matrices—such as liver, brain or gastric contents—where “matrix effects” can significantly influence the ionization of the analyte. Without the systematic use of deuterated internal standards to compensate for such effects, recovery rates can vary considerably from one protocol to another, adding a further margin of uncertainty to the comparison of concentration studies in matrices other than blood. Several studies did not specify the LLOQ for oxycodone; however, when reported, it ranged from 0.001 mg/L to 0.05 mg/L, with UPLC–MS/MS methods generally achieving the lowest LLOQs (0.005–0.0095 mg/L), whereas GC-MS and GC-NPD methods typically ranged from 0.02 to 0.05 mg/L.

### 3.4. Peripheral Blood Concentrations

**Figure 2:**
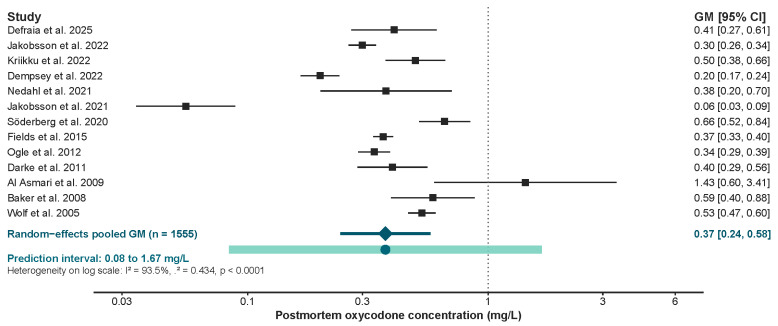
Pooled geometric mean peripheral blood oxycodone concentrations in fatal intoxication deaths [11,13,18,19,20,21,22,23,24,25,26,27,28] (squares represent study-specific geometric mean concentrations and horizontal lines indicate 95% confidence intervals. The diamond represents the pooled random-effects estimate, while the thick horizontal bar indicates the prediction interval).

**Figure 3:**
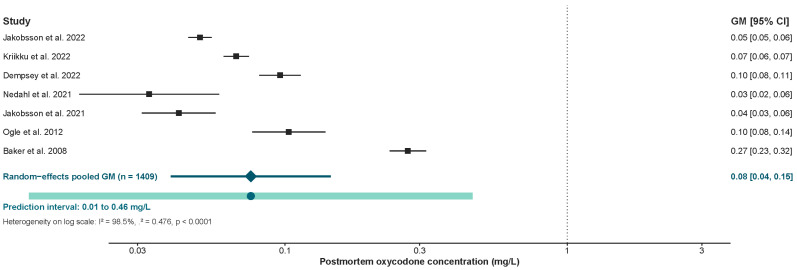
Pooled geometric mean peripheral blood oxycodone concentrations in non-intoxication deaths [19,20,21,22,23,25,27] (graphical elements are described in Figure 2).

**Figure 4:**
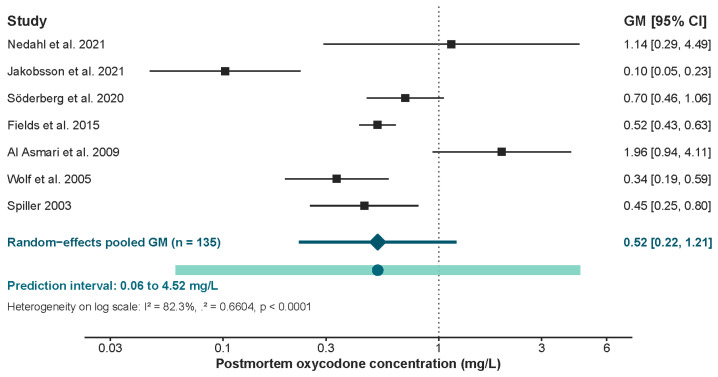
Pooled geometric mean peripheral blood oxycodone concentrations in mono-intoxication deaths [11,13,22,23,24,28,29] (graphical elements are described in Figure 2).

**Figure 5:**
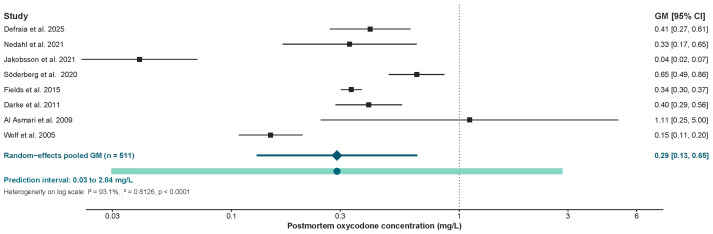
Pooled geometric mean peripheral blood oxycodone concentrations in mixed-drug intoxication deaths [11,13,18,22,23,24,26,28] (graphical elements are described in Figure 2).

The included studies adopted heterogeneous approaches for classifying the role of oxycodone in determining cause of death, and substantial overlap in postmortem concentrations was observed across forensic contexts. Consequently, quantitative syntheses were restricted to studies providing sufficiently reliable classification of fatal intoxication and non-intoxication deaths. In the primary meta-analysis, fatal intoxication cases (*n* = 1555) showed a pooled GM peripheral blood oxycodone concentration of 0.37 mg/L (95% CI: 0.24–0.58), with substantial heterogeneity across studies (I^2^ = 93.5%, *p* < 0.0001; prediction interval: 0.08–1.67 mg/L; Figure 2). Non-intoxication deaths (*n* = 1409) showed lower pooled concentrations, with a pooled GM of 0.08 mg/L (95% CI: 0.04–0.15), also with very high heterogeneity (I^2^ = 98.5%, *p* < 0.0001; prediction interval: 0.01–0.46 mg/L; Figure 3). Subgroup analyses additionally distinguished mono-intoxication and mixed-drug intoxication cases. Mono-intoxication cases (*n* = 135) showed a pooled GM concentration of 0.52 mg/L (95% CI: 0.22–1.21), with substantial heterogeneity (I^2^ = 82.3%, *p* < 0.0001; prediction interval: 0.06–4.52 mg/L; Figure 4), whereas mixed-drug intoxication cases (*n* = 511) showed lower pooled concentrations, with a pooled GM of 0.29 mg/L (95% CI: 0.13–0.65), also with substantial heterogeneity (I^2^ = 93.1%, *p* < 0.0001; prediction interval: 0.03–2.84 mg/L; Figure 5).

### 3.5. Metabolites and Metabolite-to-Parent Ratios

Data on oxycodone metabolites were reported in six studies [13,22,23,24,31,36]. Noroxycodone (NOC) and oxymorphone (OM) were the principal metabolites, although their detection frequency, analytical targets (free vs. total metabolites), and the level of quantitative detail varied across studies and matrices. NOC was the metabolite most consistently present in postmortem blood and generally occurred at higher concentrations than OM, with a median peripheral blood concentration of 0.051 mg/L, as reported in the largest dataset by Jakobsson et al. [31]. It was also found in vitreous humor and urine following oxycodone intoxication [13]. OM appeared less frequently across studies. Postmortem blood concentrations ranged from approximately 0.005 mg/L [22] to 0.048 mg/L [36], with 0.011 mg/L observed in several studies [31,36]. OM was additionally found in brain tissue and urine [13,22]. Where metabolite-to-parent ratios were reported, substantial variability emerged. Differences were associated with the presence of benzodiazepines among co-intoxicants [24], and with CYP2D6 phenotype as observed by Jakobsson et al. [31], who quantitatively described NOC/oxycodone, OM/oxycodone, and noroxymorphone/oxycodone ratios (see Table 1). Moreover, Al-Asmari et al. investigated median parent-to-metabolite ratios (oxycodone/NOC) [13].

### 3.6. Alternative Biological Matrices Other than Peripheral Blood and Postmortem Interval

A minority of the included studies investigated oxycodone concentrations in biological matrices other than peripheral blood. Seven studies provided quantitative data allowing both reporting of concentrations in specific matrices and calculation of ratios relative to peripheral blood [13,18,22,30,33,35,38], in addition to the study by Thompson et al. which reported quantitative data based on cardiac blood [36]. Data from alternative matrices were also described in selected case reports by Cone et al., although these were not systematically reported and did not allow calculation of matrix-to-blood ratios [37]. Vitreous humor (VH)/PB ratios ranged from approximately 1 [30,35,38] to 1.5 [13,18]. Central blood/PB ratios varied from around 0.30 [18] to approximately 2 [38], with intermediate values of 1.65, reported by Havig et al., and Zilg et al. [30,33]. Urine/PB ratios were consistently high (around 18), although marked variability was observed (SD ≈ 18) [18,30,38]. Additional matrices reported included liver, gastric contents, and bile [18,38], skeletal muscle [18,30], and brain [18,22]. PMI data were reported in 7 studies, mainly those investigating alternative matrices [18,30,33,36,38], as well as in the works by Spiller et al., and Mantinieks et al., which examined antemortem/postmortem concentration ratios [29,32]. Reported PMIs ranged from <24 h [30,36,38], to 8–10 days [18,30], with most cases sampled between 48 and 72 h.

## 4. Discussion

This study represents the first systematic review and quantitative meta-analysis specifically focused on postmortem oxycodone toxicology. By integrating pooled peripheral blood concentration data with evidence regarding metabolite ratios, alternative matrices, pharmacogenomic variability, and postmortem factors, the present findings emphasize the complexity and contextual nature of postmortem oxycodone interpretation. The following key points emerge for toxicologists and forensic pathologists:

- Postmortem oxycodone concentrations are highly variable, right-skewed, and substantially overlapping across forensic contexts, making their interpretation complex and multifactorial;
- Non-intoxication deaths show roughly one-fourth of the peripheral blood oxycodone concentration observed in fatal intoxications, which are mostly mixed-drug cases with generally lower concentrations than mono-intoxications, due to additive or synergistic effects of co-intoxicants;
- Postmortem oxycodone concentrations are influenced by multiple biological, forensic, and methodological factors, including polydrug exposure, opioid tolerance, prescription status, demographic characteristics, and PMI, with prolonged intervals (particularly in cases of residual gastrointestinal content) potentially enhancing postmortem redistribution effects;
- Although peripheral blood remains the reference matrix in forensic toxicology, alternative matrices (vitreous humor, liver, brain, etc.) together with metabolite-to-parent ratios provide complementary interpretative information regarding redistribution and acute versus chronic intake;
- Comprehensive forensic interpretation requires integrated evaluation of toxicological, circumstantial, and autopsy findings, supported by advanced analytical approaches such as LC–MS/MS.

A pronounced heterogeneity emerged across oxycodone-positive postmortem cases and represents one of the central findings of this review. Variability was observed not only in measured oxycodone concentrations, but also in study populations, forensic contexts, autopsy protocols, analytical methodologies, and case classification criteria.

Several factors probably contributed to this variability. First, the included populations were highly heterogeneous, encompassing therapeutic use, abuse, mixed-drug intoxications, and deaths unrelated to oxycodone toxicity. While male predominance was a relatively consistent feature across studies, other factors (including age, prescription status, polydrug exposure, and manner of death) varied substantially among oxycodone-positive cases. Abuse was more frequently reported among younger males, whereas therapeutic use predominated in older decedents, reflecting demographic and behavioral differences, as described by Kriikku et al. and Häkkinen et al. [20,34]. Häkkinen et al. further highlighted variability in the classification of oxycodone-related deaths, distinguishing concentrations between an “abuse” group and a heterogeneous “other use” category that still included a non-negligible proportion of fatal poisonings [34]. Manner of death was explicitly reported in several studies, although not systematically; overall, accidental deaths predominated among oxycodone-positive cases, with suicide and natural causes also represented a substantial proportion of fatalities [19,20,23,26,38].

Second, important analytical variability was identified across studies, with reported LLOQs ranging from 0.001 mg/L to 0.05 mg/L, thereby representing a further factor contributing to between-study variability. This discrepancy constitutes a potential source of analytical bias, as lower analytical sensitivity may have limited the detection of oxycodone in therapeutic or non-intoxication-related cases. Differences in analytical platforms, toxicological screening strategies, specimen handling, and reporting practices may have further influenced the detection of low concentrations and, consequently, some meta-analytic comparisons.

Within this heterogeneous framework, the quantitative meta-analysis nevertheless identified consistent overall trends across studies. Fatal intoxication cases generally showed higher pooled peripheral blood oxycodone concentrations than non-intoxication deaths (GM 0.37 vs. 0.08 mg/L). Similar trends were consistently reported across individual studies, including those by Kriikku et al. (0.38–0.64 vs. 0.06–0.10 mg/L), Dempsey et al. (0.46 vs. 0.15 mg/L), and Jakobsson et al. (0.30 vs. 0.05 mg/L), despite considerable variability in absolute concentration ranges and case-classification criteria [20,21,23]. Some datasets also reported markedly elevated intoxication concentrations (around 2 mg/L) [13,27], further emphasizing the broad variability observed across forensic contexts. Consistent with these findings, the wide prediction intervals observed in the meta-analyses (e.g., 0.08–1.67 mg/L for fatal intoxications and 0.01–0.46 mg/L for non-intoxication deaths) highlight the substantial overlap between interpretative categories and the marked variability expected in real-world forensic settings. Subgroup analyses further clarified intoxication patterns, showing higher oxycodone concentrations in mono-intoxication cases compared with mixed-drug intoxications (GM 0.52 vs. 0.29 mg/L). This difference likely reflects both analytical variability and pharmacological interactions, as co-administered substances may modulate oxycodone effects through additive or synergistic mechanisms, thereby reducing the concentration required to produce fatal toxicity [19,24,29,37]. Despite these overall trends, substantial overlap persisted between mono- and mixed-intoxication cases, with wide prediction intervals observed for both categories (0.06–4.52 mg/L and 0.03–2.84 mg/L, respectively), reflecting the inherent complexity of real-world forensic scenarios, as already described in the literature [28,36]. Moreover, a similar overlap exists between postmortem cases and living populations (e.g., DUID), in which oxycodone concentrations may exceed those observed in many decedents (0.68 mg/L, higher than 91% of postmortem cases) [21], likely reflecting opioid tolerance [23]. Collectively, these findings help explain the substantial between-study heterogeneity observed across all quantitative syntheses despite logarithmic modeling, with I^2^ values generally exceeding 80% and wide prediction intervals identified for all major forensic categories. Such variability was therefore not solely attributable to statistical dispersion or distributional skewness, but also reflected genuine biological variability, heterogeneous forensic populations, analytical differences, and methodological inconsistencies across postmortem studies [11]. Many of these sources of variability are intrinsically difficult to standardize in retrospective forensic research. For this reason, the present review also considered additional toxicological markers beyond peripheral blood oxycodone concentrations in order to provide complementary interpretative information. Metabolite data, reported in only a minority of studies, may help contextualize postmortem findings. Ratios such as NOC/oxycodone and OM/oxycodone can inform timing of intake, acute versus chronic use, and individual metabolic variability, including CYP2D6-mediated differences [31]. The NOC/oxycodone ratio was roughly three times lower in oxycodone-related deaths, supporting its utility in identifying acute intake. OM/oxycodone ratios < 0.075 also showed high sensitivity for acute intake, though affected by CYP2D6 activity [31]. These findings align with Al-Asmari et al., who reported high median oxycodone/NOC ratios associated with shorter survival times [13]. The detection of metabolites in alternative matrices further emphasizes that, although peripheral blood remains the primary matrix in forensic toxicology, other biological substrates can provide valuable complementary information. Vitreous humor concentrations are relatively stable and allow metabolite detection, supporting their reliability when blood is unavailable or affected by postmortem redistribution [13,18,35]. Brain, liver, and skeletal muscle, though more variable, inform tissue distribution, cumulative exposure, and potential central effects [22,30]. In addition, PMI significantly influences concentration interpretation, with early sampling (≤72 h) generally providing more reliable results, whereas longer PMIs may increase variability in central/peripheral and arterial/venous ratios [33], due to postmortem redistribution or residual gastrointestinal content, especially with extended-release formulations [18,38]. Therefore, integrating metabolite ratios, alternative matrices, and PMI data supports more robust forensic interpretation than reliance on peripheral blood concentrations alone. Nevertheless, the findings of this review should be interpreted in light of several limitations, including the retrospective nature of the included studies, heterogeneous case-classification criteria, incomplete reporting of clinical and toxicological variables, variability in analytical sensitivity, and the need to harmonize heterogeneous summary statistics for quantitative synthesis. Moreover, individual-level data were frequently unavailable, limiting more advanced analyses and preventing reliable reconstruction of concentration distributions across studies. Collectively, these findings underscore the importance of a timely and thorough autopsy and a detailed, holistic forensic interpretation when evaluating positive oxycodone toxicology, consistent with broader forensic literature emphasizing the value of comprehensive postmortem assessment in complex death investigations [39,40]. All factors must be carefully considered, as appearances can be misleading: even the presence of oxycodone at a scene does not guarantee its detection in biological matrices, especially in cases involving counterfeit tablets containing potent synthetic opioids [41,42]. From a practical forensic perspective, these considerations support the adoption of more standardized toxicological approaches and better-designed prospective studies incorporating comprehensive case-history reconstruction, appropriate postmortem sampling strategies, metabolite interpretation, and careful evaluation of postmortem redistribution, while recognizing that postmortem oxycodone interpretation remains intrinsically probabilistic because concentrations are highly variable, right-skewed, and potentially overlapping across forensic contexts.

## 5. Conclusions

The present systematic review and meta-analysis represents the first quantitative synthesis specifically focused on postmortem oxycodone toxicology. Meta-analytic results show that fatal intoxications generally exhibit higher peripheral blood oxycodone concentrations than non-intoxication deaths. However, substantial heterogeneity and wide meta-analytic prediction intervals preclude the definition of fixed concentration cut-offs. These findings highlight the need to evaluate oxycodone’s role in death within a probabilistic forensic framework integrating circumstantial evidence, time of sampling, metabolite ratios, and data from alternative biological matrices. Future research based on standardized forensic protocols, harmonized reporting practices, advanced analytical approaches (particularly LC–MS/MS), and well-characterized case series may improve the reliability and consistency of postmortem oxycodone interpretation, support forensic decision-making, and strengthen public health monitoring.

## Figures and Tables

**Table 1:** Characteristics of included studies and sampled matrices in postmortem human oxycodone cases.

Study (First Author, Year, Journal) [Reference]	Country/Study Period	Study Population *n*/N (%Male; Age Mean (SD)	Case Classification: Cause of Death (*n*, %) and/or Manner of Death (*n*, %)	PMI at Sampling	Matrix (Biological Samples, Tissues)
Defraia B et al. 2025, Drug Test Anal. [18]	Italy/NA	2/2 (100%; 23 (6))	Fatal intoxications (100%) after ingestion of multiple OxyContin tablets	72 h (t1) and 10 d (t2)	PB, CB, VH, U, GC, bile, solid tissues (liver, brain, skeletal muscle, kidney, adipose tissue)
Jakobsson G et al. 2022, Eur J Drug Metab Pharmacokinet [19]	Sweden/2012–2018	1081/1236 (66%; 55 (17))	Intoxications 451 (42%) Non-intoxications 630 (58%)	NA	PB
Kriikku P et al. 2022, Int J Legal Med [20]	Finland/2016–2019	486/486 (56%; 68)	Intoxications 121 (42%) Non-intoxications 365 (58%) Suicide 37(8%); Accidental 67(14%); Natural 365(75%); Undetermined 157(15%)	NA	PB
Dempsey SK et al. 2022, J Anal Toxicol. [21]	USA (Houston, TX)/2014–2020	309/4000 (NA)	Intoxications 189 (61%) Non-intoxications 120 (39%)	NA	PB
Havig SM et al. 2022, J Anal Toxicol. [30]	Norway/2013–2016	8/54 (NA)	Suspicion of positive toxicological findings by pathologists	0–8 d	PB, CB, VH, PF, skeletal muscle (PMM, VLM),
Jakobsson G et al. 2021, J Anal Toxicol. [23]	Sweden/2018	189/189 (64%; 57 (14))	Mono-intoxications 20 (10%) Mixed-intoxications 48 (25%) Non-intoxications 93 (50%) Other intoxications 28 (15%)	NA	PB
Jakobsson G et al. 2021, Forensic Sci Int Genet. [31]	Sweden/2018	174/189 (64%; 57 (20))	CYP2D6 phenotype groups: PM + IM (74), EM + UM (100) Suicide 38(22%); Accidental 45(26%); Natural 56(32%); Undetermined 35(20%)	NA	PB
Nedahl M et al. 2021, J Anal Toxicol. [22]	Denmark/2015–2019	51/210 (NA)	Mono-intoxications 2 (4%) Mixed-intoxications 21 (41%) Non-intoxications 28 (55%)	NA	PB and Brain tissue
Mantinieks D et al. 2021, J Anal Toxicol. [32]	Australia/2009–2017	33/811 (NA)	NA	31–48 h	PB
Söderberg C et al. 2020, Forensic Sci Int [11]	Sweden/1992–2010	49/3767 (70%; 54)	Mono-intoxications 19 (39%) Mixed-intoxications 26 (53%) Non-intoxications 4 (8%)	75–115 h	PB
Zilg B et al. 2017, Forensic Sci Int [33]	Sweden/2012–2016	4/48 (74%; 41 (14))	All suspected intoxications	0–216 h	Venous PB (ext. iliac, jugular) arterial PB (carotid), CB (LH, RH)
Fields MD et al. 2015, J Forensic Sci [24]	USA (West Virginia)/2005–2010	270/877 (70%; 39 (14))	Mono-intoxications 48 (18%) Mixed-intoxications 222 (72%)	NA	PB
Häkkinen M et al. 2014, Forensic Sci Int [34]	Finland/2010–2011	598/2088 (64%; 68 (18))	Abuse-deaths 41 (7%)—fatal poisoning in 39% Other use-deaths 557 (93%)—fatal poisoning in 3%	NA	PB
Ogle A et al. 2012, Forensic Sci Int [25]	USA (Florida)/2009	154/154 (58%; 42 (13))	Mono-intoxications 10 (6%) Mixed-intoxications 106 (69%) Non-intoxications 38 (25%)	NA	PB
Darke S, et al. 2011, J Forensic Sci. [26]	Australia (NSW)/1999–2008	70/70 (59%; 49)	Mixed-intoxications (100%) Suicide 15 (21%)	NA	PB
Knittel JL et al. 2009, J Anal Toxicol. [35]	USA/NA	NA	NA	NA	Blood (not specified P or C); VH
Al-Asmari A et al. 2009, J Anal Toxicol. [13]	Scotland/2009	10 (50%; 44 (17))	Mono-intoxications 4 (40%) Mixed-intoxications 5 (50%) Non-intoxications 1 (10%)	NA	PB, CB, U, VH, GC
Thompson JG et al. 2008, J Anal Toxicol. [36]	USA (Hennepin County ME, 2000–2005)	67/67 (NA)	Mono-intoxications 7 (10%) Mixed-intoxications 23 (35%) Non-intoxications 37 (55%)	24–72 h	CB
Baker DD et al. 2008, J Anal Toxicol. [27]	USA (Cuyahoga County, Ohio)/1998–2003	190/445 (64.7%; 53)	Intoxications 55 (29%) Non-intoxications 135 (71%)	NA	PB
Wolf BC et al. 2005, J Forensic Sci. [28]	USA (Palm Beach County)/NA	172/172 (77%; 41)	Mono-intoxications 18 (10%) Mixed-intoxications 117 (68%) Non-intoxications 37 (22%; of which trauma 23 and natural 14)	NA	PB
Cone EJ et al. 2004, J Anal Toxicol. [37]	USA/ 1999–2002	839/1243 (NA)	Mono-intoxications 27 (3%) Mixed-intoxications 746 (89%) Non-intoxications 66 (8%)	NA	Blood (not specified the anatomical site of sampling)
Spiller HA. 2003, J Forensic Sci. [29]	USA (10 counties)/2000–2001	71/88 (72%; 39 (11))	Mono-intoxications 24 (34%) Mixed-intoxication 47 (66%)	<24 h	PB
Anderson DT et al. 2002, J Anal Toxicol. [38]	USA (Los Angeles County)/1995–2001	36/67 (50%; 44 (11))	OxyContin CR identified Accidental 20 (55.6%); Suicides 10 (27.8%), Natural: 4 (11.1%); Undetermined: 2 (5.6%)	24–72 h	PB, CB, U, VH, bile, GC, Liver

Abbreviations: CB, central blood; d, days; GC, gastric content; h, hours; LH, left heart blood;; N, total study population; *n*, number of oxycodone-positive cases; NA, not available; PB, peripheral blood; PF, pericardial fluid; PMI, postmortem interval; PMM, psoas major muscle; RH, right heart blood; U, urine; VH, vitreous humor; VLM, vastus lateralis muscle; Y, years.

**Table 2:** Toxicological results and postmortem findings in human oxycodone cases.

Study (First Author, Year, Journal) [Reference]	Analytical Method; LLOQ (mg/L)	Oxycodone Concentration (mg/L or mg/kg): Mean (SD) or Median (Range) or Ratios	Oxycodone Metabolites	Co-Intoxicants	Histopathological Findings
Defraia B et al. 2025, Drug Test Anal. [18]	Enzyme immunoassay and UPLC–MS/MS (screening) LC–HRMS (confirmation); LLOQ NA	**PB (t1) 0.42 (0.08)**; PB (t2) 3.62 (1.52); VH (t1) 0.64 (0.23); CB (t2) 1.09 (0.69); U (t1) 7.5 (2.5); U (t2) 6.5 (2.5); Bile (t2) 3 (2); GC 27 (23); Liver > 25 (24); Brain 0.78 (0.01); Skeletal Muscle 0.59 (0)	NA	BDZs (alprazolam, diazepam, bromazepam); ethanol	Generalized congestion; myocardial contraction band necrosis; perivascular fibrosis (case 1); pulmonary edema; no traumatic injuries
Jakobsson G et al. 2022, Eur J Drug Metab Pharmacokinet [19]	NA	Intoxications: **0.30 (0.005–83)** Non-intoxications: **0.05 (0.005–7.1)**	NA	High prevalence of CNS depressants (BDZs, alcohol, other PDI drugs)	NA
Kriikku P et al. 2022, Int J Legal Med [20]	GC–MS (screening and confirmation); LLOQ 0.02	Intoxications-abuse: **0.38 (0.02–47)** Intoxications non-abuse: **0.64 (0.04–48)** Non-intoxications: **0.06–0.10 (0.002–5.5)**	NA	Multiple drugs in intoxications (but specific co-intoxicants NA)	Variable; no specific oxycodone-related histopathological pattern
Dempsey SK et al. 2022, J Anal Toxicol. [21]	LC–TOF-MS (screening) GC–MS, GC–MS/MS or LC–MS/MS (confirmation); LLOQ 0.005	Intoxications: **0.46 (0.01–11)** Non-intoxications: **0.15 (0.01–0.75)**	NA	NA	NA
Havig SM et al. 2022, J Anal Toxicol. [30]	UPLC–MS/MS (screening and confirmation); LLOQ 0.0095	**PB: 0.23 (0.018–0.47); CB: 0.24 (0.018–1.1)**; PF: 0.29 (0.34–2.0); PMM: 0.17 (0–0.70); VLM: 0.15 (0–0.54); VH: 0.16 (0.027–0.62); VH/PB 1.2	NA	Multiple drugs (but specific co-intoxicants NA)	No specific oxycodone-related histopathological pattern
Jakobsson G et al. 2021, J Anal Toxicol. [23]	LC-MS/MS (validated, protein precipitation, Waters UPLC Xevo TQD); LLOQ 0.005	Mono-intoxications: **0.55 (0.13–11)** Mixed-intoxications: **0.34 (0.021–13)** Non-intoxications: **0.42 (0.005–7.4)**	NOC (98%), OM (36%), NOM (44%)	High prevalence of CNS depressants (BDZs, alcohol)	NA
Jakobsson G et al. 2021, Forensic Sci Int Genet. [31]	LC–TOF-MS; CYP2D6 genotyping (*3, *4, *5, *6, copy number variation via ddPCR)	Total **0.099 (0.006–12.7)** PM + IM **0.15 (0.009–1.9)** EM + UM **0.072 (0.006–12.7)**	**NOC 0.051 (0.005–1.9)** **OM 0.011 (0.005–0.044)** **NOM 0.009 (0.005–0.079)** NOC/OC 0.54 (0.076–2.2); OM/OC 0.045 (0.004–0.66); NOM/OC 0.071 (0.006–0.91)	Multiple drugs present in >70% mixed intoxications (but specific co-intoxicants NA)	NA
Nedahl M et al. 2021, J Anal Toxicol. [22]	LC–MS, validated, SPE; quantitative confirmation with triple quadrupole MS; LLOQ NA	Mono-intoxications: **mean 1.43 (1.22)** Mixed -intoxications: **0.33 (0.094–5.5)** Non-intoxications: **0.033 (0.006–0.31);** Brain: 0.56 (0.15–1.8); Brain/blood ratio: 1.8 (0.11–6.0)	OM detected qualitatively in 6/51 (12%) cases: 2 cases in both PB and brain, 3 only in PB, 1 only in brain	Other CNS depressants	Not specific oxycodone-related histopathological pattern
Mantinieks D et al. 2021, J Anal Toxicol. [32]	LC-MS/MS; LLOQ NA	**0.071 (0.010–1.9)** median PostMortem/AnteMortem ratios 1.0	NA	NA	NA
Söderberg C et al., 2020, Forensic Sci Int [11]	GC-NPD; LLOQ 0.05	Mono-intoxications: **0.70 (0.50–5.22)** Mixed -intoxications: **0.65 (0.40–2.60)** Non-intoxications: **0.10 (0.10–0.40)**	NA	NA	NA
Zilg B et al. 2017, Forensic Sci Int [33]	LC–TOF (screening) LC-MS/MS or GC-MS (confirmation); LLOQ NA	CB/PB 1.6 Arterial blood/Venous blood 1.7	NA	NA	NA
Fields MD et al. 2015, J Forensic Sci [24]	Immunoassay (screening) LC-MS, LC-MS/MS or GC-MS (confirmation); LLOQ NA	Mono-intoxications: median **0.52** Mixed-intoxications: median **0.40, 0.27, 0.26 (respectively with 2, 3 or with 4 co-intoxicants)**	OC/OM (78) 3.53–10.48 (varied with BDZ presence, no significant differences)	BDZs (alprazolam, diazepam), other drugs	No specific oxycodone-related histopathological pattern
Häkkinen M et al. 2014, Forensic Sci Int [34]	NA	Abuse-deaths: **0.24 (0.02–1.5)** Other-use-deaths: **0.08 (0.01–10)**	NA	Other opioids; ethanol	NA
Ogle A et al. 2012, Forensic Sci Int [25]	Immunoassay (screening) GC-MS (confirmation); LLOQ NA	Intoxications: **0.48 (0.49)** Non- intoxications: **0.16 (0.19)**	NA	Ethanol, BDZs, antidepressants, cocaine, beta-blockers, methadone, other drugs	NA
Darke S, et al. 2011, J Forensic Sci. [26]	Immunoassay (screening) GC or HPLC (confirmation); LLOQ 0.05	**0.40 (0.06–53)**	NA	BDZs, ethanol, other opioids, antidepressants	NA
Knittel JL et al. 2009, J Anal Toxicol. [35]	Immunoassay DRI EMIT (screening) GC–MS (confirmation); LLOQ 0.05	Blood: **0.103–0.768;** VH < 0.05–0.945; VH/Blood 1.16 (0.12–3.26)	NA	NA	NA
Al-Asmari A et al. 2009, J Anal Toxicol. [13]	LC-MS/MS; LLOQ 0.001	PB: Mono-intoxications: **2.38 (1.44)** Mixed-intoxications: **2.44 (2.55)** Non-intoxications: **0.09**; U (mono) 26.6; U (mixed) 16.76; VH (mono) 1.365	NOC (98%) median 0.5; OM mono (36%): median 0.01; OM mixed (39%) median 0.06	Ethanol, BDZs (diazepam, temazepam, levomepromazine), other prescribed drugs	Not specific oxycodone-related histopathological pattern (intact tablets recovered in stomach in 4 cases)
Thompson JG et al. 2008, J Anal Toxicol. [36]	GC–MS after SPE and derivatization with BSTFA + TMCS; LLOQ 0.05	Mono-intoxications: **mean 1.06, median 0.824 (0.27–3.39)** Mixed-intoxications: **mean 0.820, median 0.47 (0.014–3.8)** Non-intoxications: **mean 0.33, median 0.15 (0.017–1.3)**	OM 0.011–0.048 (in some cases)	Ethanol, BDZs, antidepressants, cocaine, beta-blockers, acetaminophen, methadone, amphetamines, other drugs	Not specific oxycodone-related histopathological pattern
Baker DD et al. 2008, J Anal Toxicol. [27]	GC-MS; LLOQ 0.05	Intoxications: **mean 1.86 (5.56), median 0.56 (0.01–36.54)** Non-intoxications: **mean 0.40 (0.43), median 0.26 (0.02–2)**	NA	Multiple drugs present in mixed intoxications	NA
Wolf BC et al. 2005, J Forensic Sci. [28]	NA	Mono-intoxications: **0.69 (0.21–4.71)** Mixed-intoxications: **0.72 (0.025–17.5)** Non-intoxications: **0.62**	NA	BDZs (alprazolam), cocaine	NA
Cone EJ et al. 2004, J Anal Toxicol. [37]	Immunoassay (screening); GC-MS (confirmation); LLOQ NA	Mono-intoxications **1.63 (2.55)** Mixed-intoxications **0.78 (1.37)** Non- intoxications: **0.33 (0.41)**	NA	Multiple drugs present in mixed intoxications (but specific co-intoxicants NA)	NA
Spiller HA. 2003 J Forensic Sci. [29]	GC (confirmation); LLOQ 0.05	Mono-intoxications: **mean 0.43 (0.12–8)** Mixed-intoxications: **mean 0.20 (0.34 for hydrocodone alone; 0.18 ≥ 2 co-intoxicants)**	NA	Ethanol, amitriptyline, methadone, codeine, propoxyphene, acetaminophen	NA
Anderson DT et al. 2002, J Anal Toxicol. [38]	ELISA or radioimmunoassay (screening), GC-NPD + GC-MS (confirmation); LLOQ NA	**CB 0.12–46; PB 0.10–13**; U 2.5–122; Bile 0.19–49; VH 0.24–0.82; GC 0.06–119; Liver 0.11–6.1 mg/kg	NA	NA	No specific oxycodone-related histopathological pattern

Note: The asterisk (*) denotes the standard star-allele nomenclature used in pharmacogenetics. Specifically, CYP2D6 *3, *4, *5, and *6 are variant alleles associated with reduced or absent enzyme activity. Abbreviations: BDZs, benzodiazepines; CNS, central nervous system; CYP2D6, cytochrome P450 2D6; ddPCR, droplet digital polymerase chain reaction; ELISA, enzyme-linked immunosorbent assay; GC–MS, gas chromatography–mass spectrometry; GC–NPD, gas chromatography–nitrogen phosphorus detector; LC–MS/MS, liquid chromatography–tandem mass spectrometry; LLOQ, lower limit of quantification; NA, not available; NOC, noroxycodone; NOM, noroxymorphone; OC, oxycodone; OM, oxymorphone; PMM, psoas major muscle; SPE, solid-phase extraction; TOF, time-of-flight; TQD, triple quadrupole detector; UPLC, ultra-performance liquid chromatography.

## Data Availability

The original contributions presented in this study are included in the article/Supplementary Material. Further inquiries can be directed to the corresponding author.

## Supplementary Materials

The following supporting information can be downloaded at: https://www.mdpi.com/article/10.3390/molecules31122051/s1.

## Abbreviations

The following abbreviations are used in this manuscript:

BDZs	Benzodiazepines
CB	Central blood
CNS	Central nervous system
CSF	Cerebrospinal fluid
ELISA	Enzyme-linked immunosorbent assay
GC	Gastric content
GC–MS	Gas chromatography–mass spectrometry;
GC–NPD IQR	Gas chromatography–nitrogen phosphorus detector;
IQR	Interquartile ranges
LC–MS/MS	Liquid chromatography–tandem mass spectrometry;
LLOQ	Lower limit of quantification
LH	Left heart (blood)
NOC	Noroxycodone
NOM	Noroxymorphone
OC	Oxycodone
OM	Oxymorphone
PB	Peripheral blood
PMI	Postmortem interval
SD	Standard deviation
SPE	Solid-phase extraction
TOF	Time-of-flight
TQD	Triple quadrupole detector
U	Urine
UPLC	Ultra-performance liquid chromatography
VH	Vitreous humor
