# Supplementary material for: Postmortem Oxycodone Toxicology: A Systematic Review and Meta-Analysis of Concentrations and Interpretative Markers
Source: Molecules. 2026 Jun 11;31(12):2051. doi: 10.3390/molecules31122051 (PMC13305030; doi:10.3390/molecules31122051)
Supplement: Supplementary file 1 [file molecules-31-02051-s001.zip › molecules-4256525-supplementary.pdf]

SUPPLEMENTARY MATERIAL

Supplementary Table S1. PRISMA 2020 Checklist– page 2

Supplemental Scopus search string – page 4

Supplementary Table S1. PRISMA 2020 Checklist.

Section/Topic	Item	Checklist item	Location reported in manuscript
Title	1	Identify the report as a systematic review.	Title, lines 1–3
Abstract	2	See PRISMA abstract checklist.	Abstract, lines 12–42
Rationale	3	Describe rationale in context of existing knowledge.	Introduction, lines 46–106
Objectives	4	Provide explicit objectives/questions.	Introduction, lines 100–106
Eligibility criteria	5	Specify inclusion/exclusion criteria and grouping.	Methods, lines 130–156
Information sources	6	Specify all information sources and search dates.	Methods, lines 111–114
Search strategy	7	Present full search strategy.	Methods, lines 119–128; Supplementary Material
Selection process	8	Describe study selection methods and reviewers.	Methods, lines 130–142
Data collection process	9	Describe data extraction process.	Methods, lines 142–156
Data items	10a	Define all outcomes sought.	Methods, lines 151–156
Data items	10b	Define other variables collected.	Methods, lines 142–151
Risk of bias assessment	11	Specify methods used to assess risk of bias.	Methods, lines 146–151; lines 178-180
Effect measures	12	Specify effect measures used.	Methods, lines 160–178
Synthesis methods	13a	Describe processes used to decide study eligibility for synthesis.	Methods, lines 130–142
Synthesis methods	13b	Describe methods to prepare data for synthesis.	Methods, lines 160–170
Synthesis methods	13c	Describe methods used to tabulate/display results.	Tables 1–2; Figures 1–5
Synthesis methods	13d	Describe synthesis methods and heterogeneity assessment.	Methods, lines 170–183
Synthesis methods	13e	Describe methods exploring heterogeneity.	Subgroup analyses, lines 153-156; 175-178
Synthesis methods	13f	Describe sensitivity analyses.	No formal sensitivity or robustness analyses reported
Reporting bias assessment	14	Describe methods assessing reporting bias.	Methods, lines 178–180
Certainty assessment	15	Describe certainty/confidence assessment.	Not performed
Study selection	16a	Describe search/selection results.	Results, lines 185–193; Figure 1
Study selection	16b	Cite excluded studies and reasons.	Selection process summarized in Figure 1; no separate exclusion table

Study characteristics	17	Present characteristics of included studies.	Table 1
Risk of bias in studies	18	Present risk of bias assessments.	Narrative domain-based assessment reported, Methods lines 146–151
Results of individual studies	19	Present summary statistics/effect estimates.	Table 2; Figures 2–5
Results of syntheses	20a	Summarise characteristics and RoB among studies.	Results, lines 217–311
Results of syntheses	20b	Present statistical synthesis results.	Results, lines 248–260; Figures 2–5
Results of syntheses	20c	Present investigations of heterogeneity.	Results, lines 250-260; Discussion, lines 340–402
Results of syntheses	20d	Present sensitivity analyses.	No formal sensitivity analyses reported
Reporting biases	21	Present assessment of reporting bias.	Not formally assessed
Certainty of evidence	22	Present certainty assessment.	Not performed
Discussion	23a	Interpretation in context of evidence.	Discussion, lines 312–442
Discussion	23b	Discuss limitations of included evidence.	Discussion, lines 423–429
Discussion	23c	Discuss limitations of review processes.	Discussion, lines 423–429
Discussion	23d	Discuss implications for practice/research.	Discussion and Conclusions, lines 436–455
Registration and protocol	24a	Provide registration details.	No registration reported
Registration and protocol	24b	Indicate protocol access.	Methods, lines 111–112
Registration and protocol	24c	Describe amendments to protocol.	Not applicable / not reported
Support	25	Describe sources of support.	Funding, line 461
Competing interests	26	Declare competing interests.	Conflicts of Interest, line 464
Availability of data	27	Report availability of materials/data/code.	Methods, lines 156–158

Supplemental Scopus search string

The complete search string adapted for Scopus was as follows:

(oxycodone OR noroxycodone OR oxymorphone OR noroxymorphone) AND (postmortem OR post-mortem OR autopsy OR "drug-related death" OR overdose OR poisoning OR fatal) AND ("peripheral blood" OR "femoral blood" OR "central blood" OR "cardiac blood" OR blood OR "vitreous humor" OR urine OR "gastric content" OR "intraosseous fluid" OR brain OR liver OR kidney OR muscle OR adipose OR bone) AND ("LC-MS/MS" OR "liquid chromatography tandem mass spectrometry" OR "gas chromatography" OR GC-MS OR LC-MS OR quantification OR concentration).
